# Selected risk factors for atherosclerosis in children and their parents with positive family history of premature cardiovascular diseases: a prospective study

**DOI:** 10.1186/s12887-018-1102-2

**Published:** 2018-04-03

**Authors:** Elzbieta Pac-Kozuchowska, Paulina Krawiec, Ewelina Grywalska

**Affiliations:** 1 Department of Paediatrics and Gastroenterology, Medical University of Lublin, Gebali 6 Street, 20-093 Lublin, Poland; 2 Department of Clinical Immunology and Immunotherapy, Medical University of Lublin, Chodzki 4a Street, 20-093 Lublin, Poland

**Keywords:** Atherosclerosis, Cardiovascular risk, Lipid profile

## Abstract

**Background:**

The aim of the study was to evaluate serum parameters of lipid metabolism, homocysteine, soluble adhesion molecules and common carotid artery wall thickness in children from families with early symptoms of atherosclerosis.

**Methods:**

The first stage included 137 pairs of mothers and newborns, and the second 18 children from the same group (age 18-30 months) and their parents (age 21-46 years) with a history of premature coronary artery disease (CAD)*,* as well as 12 age- and sex-matched controls.

**Results:**

During the first stage, inverse correlations were found between birthweight, cord blood concentrations of triglycerides (TG), VLDL cholesterol and apolipoprotein B (Apo B). Serum concentrations of total cholesterol (TC), apolipoprotein A1 (Apo A1), LDL and HDL cholesterol and were significantly higher in female than in male newborns. During the second stage, children from families with a history for premature CAD were shown to present with significantly higher serum concentrations of TG, VLDL cholesterol and lipoprotein A (Lp(a)) than the controls. Furthermore, their TC correlated positively with vascular cell adhesion molecule-1 (Rs=0.717, p<0.05) and intracellular adhesion molecule-1 (sICAM-1) levels (Rs=0.833, p<0.05). Moreover, positive correlations were found between maternal carotid intima media thickness (IMT) and TC (Rs=0.831, p<0.01), as well as between paternal IMT and Apo B (Rs=0.692, p<0.05), TG and sICAM-1 (Rs=0.912, p<0.01), TG and sE-selectin (Rs=0.678, p<0.05).

**Conclusions:**

Serum Lp(a) may serve as a maker of cardiovascular risk in children and adolescents.

## Background

Although cardiovascular diseases based on atherosclerosis, like ischemic heart disease or stroke, manifest in adulthood, there is a compelling evidence that the process of atherosclerosis begins in childhood and progresses to measureable vascular changes in adulthood [1, 2].

The pathogenesis of atherosclerosis has undergone many changes over the last decades. The traditional viewpoint established atherosclerosis as a localized cholesterol storage disease with flow-limiting arterial stenosis [3]. We currently understand atherosclerosis as a chronic inflammatory process of arterial wall resulting from interplay of lipid metabolism imbalance, maladaptive immune response and genetic alterations [3–5].

Traditionally cardiovascular modifiable risk factors included cigarette smoking, hypercholesterolemia, hypertension and diabetes [6–8]. Approximately 20% of cardiovascular events occur in the absence of above mentioned conventional risk factors [9].

Intense investigations on novel risk factors are conducted to define cardiovascular risk in individuals without traditional risk factors. Recent studies have shown several important non-traditional risk factors for atherosclerosis including hs-CRP, homocysteine, small dense low density lipoprotein particles (small dense LDL), oxidized low density lipoprotein (oxy-LDL), apolipoprotein A1 and B (Apo A1 and Apo B), lipoprotein a (Lp(a)), fibrinogen and triglyceride-rich lipoprotein remnants [10].

Non- modifiable atherosclerosis risk factors include age, gender and family history of premature cardiovascular diseases (CVD). The growing body of evidence indicates that the family history of premature coronary artery disease (CAD) is an independent risk factor for CVD and significant predictor for CAD [11–14]. According to The Third Report of the Expert Panel on Detection, Evaluation, and Treatment of the High Blood Cholesterol in Adults (ATP III) family history of premature CAD, specify as myocardial infarction or sudden death before 55 years of age in father or other male first-degree relative, or before 65 years of age in mother or other female first-degree relative) was found as the major risk factor of CAD [15]. Accumulation of family history of premature CVD with obesity, hypertension and diabetes is often accompanied. Thus, lifestyle intervention including dietary modification, increased physical activity, weight control and smoking cessation may be more efficient when undertaken by the entire family than the individual due to built-in support mechanism of the family [16].

The aim of the study was to evaluate the parameters of lipid metabolism, i.e. triglycerides (TG), total cholesterol (TC) and its fractions: HDL, LDL and VLDL cholesterol, apolipoprotein A1 and B (Apo A1 and Apo B), lipoprotein(a) (Lp(a)), homocysteine, soluble adhesion molecules (sICAM-1, sVCAM-1 and sE-selectin) in the blood serum and the thickness of common carotid artery wall (IMT) in children from families in which early symptoms of atherosclerosis.

## Methods

The study was performed in two stages. During the first stage, the study group was composed of 137 pairs of mothers and their newborns (74 girls and 63 boys). To the study group we recruited healthy newborns, from pregnancies free of complications, born at term by spontaneous vaginal delivery with Apgar score 8-10 points at first minute after birth. The family history of cardiovascular diseases (ischemic heart disease, stroke) and risk factors for atherosclerosis like obesity, hypertension, type 2 diabetes mellitus and hypercholesterolemia was undertaken from mothers. There were selected 41 of 137 families (29.9%) with risk factors for atherosclerosis.

In all 137 pairs of mothers and newborns we assessed nutritional status and serum concentrations of TG, TC, HDL, LDL, VLDL, Apo A1, Apo B and Lp(a).

The prospective evaluation of all newborns and their parents was planned in 18 to 30 months after birth. The information about the next stage of the study was sent to all 137 families participated in the first stage of the study. However, only 41 families volunteered to the second stage of the study. In that group we selected 29 families with risk factors for atherosclerosis and 18 with positive history for premature CAD. We presumed that parents who participated in second stage of the study were aware of the cardiovascular disease danger.

Ultimately, in the second stage of the study we included 18 children (8 girls and 10 boys) aged 18 to 30 months and their parents (aged 21-46 years) with positive history for premature coronary artery disease*.* The control group consisted of 12 age- and sex-matched children without a family history of CAD. In all children and their parents we assessed serum concentrations of TG, TC, HDL, LDL, VLDL, Apo A1, Apo B, Lp(a), homocysteine (Hcy), intracellular adhesion molecule-1 (sICAM-1), vascular cell adhesion molecule-1 (sVCAM-1) and sE-selectin.

The blood samples were taken fasting from the cord blood in newborns and from the ulnar vein in children and their parents. The serum the concentration of TG, TC and HDL was determined by the Cormay reagents and the Cobas Mira S analyzer. The concentration of Apo A1, Apo B and Lp(a) was determined by immunoturbidimetric method (Roche and Human). The concentration of homocysteine was determined by ELISA method using Axis Homocysteine EIA Package Insert by Axis-Shield AS. The concentrations of sICAM-1, sVCAM-1 and sE-selectin were determined by ELISA method, with the application of kits human sICAM-1 BMS201, human sVCAM-1 BMS232, human sE-selectin BMS205 (Bender Med Systems Diagnostics GmbH). The concentration of LDL and VLDL was defined by using the formula by Friedewald [17].

**Fig. 1:**
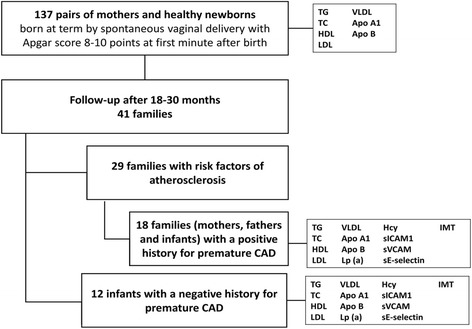
Schematic diagram of the study design

To identify early atherosclerotic changes common carotid intima and media combined layers thickness (IMT) was measured with an ATL 3500 ultrasound system with a 12 MHz linear transducer. The IMT was evaluated both on the right and left side, no significant differences were noticed thus the mean IMT value was calculated. A schematic diagram of the study design is presented at Fig. 1.

The statistical analysis of the results was made with application of Statistica 5.1 PL software. The distribution of studied data sets were checked up by Shapiro-Wilk test. The distribution of the examined parameters was skewed, thus Mann Whitney U test was applied for the analysis of the differences between two groups or Kruskal-Wallis ANOVA and multiple comparisons of mean ranks (as post-hoc analysis) for the analysis of differences between more than two groups. The Spearman R correlation test was used to assess the relationships between the parameters tested. The p values of p<0.05 were considered statistically significant.

The written informed consent to this research study was obtained from parents. The paper has been approved by Medical University Bioethical Committee (number KE-0254/74/2009).

## Results

During the first stage of the study, we recruited 137 pairs of healthy mothers and their newborns. Newborns included 74 girls (54%) and 63 boys (45%), with the mean birth weight 3268.0±529.4 g and mean body length 54.4±3.1cm. The mean gestational age was 39.1±1.5 weeks.

Table 1 presents the lipid profile and apolipoprotein concentration in newborns and their mothers.

**Table 1:** Lipid and apolipoprotein concentrations (means ± SD) in newborns’ cord blood and mothers’ serum

Parameter	Newborns	Mothers
TG (mg/dl)	58.75±66.72	221.99±89.58
TC (mg/dl)	65.05±21.39	263.58±58.01
HDL (mg/dl)	19.63±8.40	64.23±15.86
%HDL	30.74±9.27	25.20±7.48
LDL (mg/dl)	34.12±14.08	155.14±49.44
VLDL (mg/dl)	11.46±9.93	48.83±17.83
Apo A1 (mg/dl)	86.50±18.60	232.02±39.07
Apo B (mg/dl)	37.90±17.70	149.00±41.03

The results, as shown in Table 2, indicated no significant differences in newborns’ lipids parameters according to family history for cardiovascular diseases.

**Table 2:** Lipid and apolipoprotein concentrations (means ± SD) in newborns’ cord blood according to family history of CVD

Parameter	Positive history (n=41)	Negative history (n=96)	p
TG (mg/dl)	54.73±45.10	60.47±73.67	ns
TC (mg/dl)	63.39±16.67	65.76±22.98	ns
LDL (mg/dl)	33.15±11.49	34.54±14.96	ns
VLDL (mg/dl)	11.31±8.97	11.52±10.26	ns
HDL (mg/dl)	19.25±6.49	19.79±9.04	ns
Apo A1 (mg/dl)	87.80±16.78	90.28±19.18	ns
Apo B (mg/dl)	36.85±15.25	38.41±18.65	ns

ns – non-significant

In the second stage of the study volunteered 18 families with positive family history for premature CAD.

The results, as shown in Table 3, showed significantly higher serum concentration of TG, VLDL and Lp(a) in children aged 18 to 30 months from families with positive history for premature CAD compared to controls. Table 4 compares the serum lipid profile, Apo A1, Apo B, sVCAM-1, sICAM-1, Hcy and sE-selectin concentration and IMT in mothers and fathers of subjects.

**Table 3:** Comparison of selected risk factors in 18- to 30-month±-old children with a positive history for premature CAD and controls (means ± SD); 2^nd^ stage of the study

Parameter	Positive history (n=18)	Negative history (n=12)	p
TG (mg/dl)	105.44±69.04	83.03±29.26	<0.05
TC (mg/dl)	165.56±30.99	167.83±33.15	ns
HDL (mg/dl)	55.46±14.28	47.35±11.92	ns
LDL (mg/dl)	88.96±37.14	103,87±32.30	ns
VLDL (mg/dl)	21.09±13.81	16.62±5.85	<0.05
Apo A1 (mg/dl)	154.22±18.73	147.33±19.90	ns
Apo B (mg/dl)	73.11±18.82	77.58±17.59	ns
Lp (a) (mg/dl)	18.27±26.11	5.89±3.65	<0.05
Hcy (μmol/L)	12.20±2.46	10.67±2.41	ns
sVCAM-1 (ng/ml)	1616.11±561.92	1516.13±501.75	ns
sICAM-1 (ng/ml)	391.15±95.28	384.31±57.96	ns
sE-selectin (ng/ml)	58.37±17.17	72.57±38.16	ns
IMT (mm)	0.43±0.04	0.39±0.06	ns

ns – non-significant

**Table 4:** Selected risk factors for atherosclerosis in parents (means ± SD); 2^nd^ stage of the study

Parameter	Mothers (n=18)	Fathers (n=18)
TG (mg/dl)	74.56±25.59	160.00±84.40
TC (mg/dl)	177.89±34.28	180.56±23.82
HDL (mg/dl)	67.77±19.94	51.07±15.26
LDL (mg/dl)	95,21±26.80	97.49±33.00
VLDL (mg/dl)	14.91±5.12	32.00±16.88
Apo A1 (mg/dl)	189.56±33.71	162.89±15.73
Apo B (mg/dl)	70.00±16.24	106.89±43.78
Lp (a) (mg/dl)	29.60±34.48	15.43±14.47
Hcy (μmol/L)	11.63±4.19	15.07±4.26
sVCAM-1 (ng/ml)	952.98±249.80	930.41±492.07
sICAM-1 (ng/ml)	294.50±62.13	363.93±134.46
sE-selectin (ng/ml)	29.28±19.44	34.08±37.51
IMT (mm)	0.59±0.2	0.61±0.10

In children with a positive family history for premature CAD were found positive correlations between TC and sVCAM-1 (Rs=0.717, p<0.05), and TC and sICAM-1 (Rs=0.833, p<0.05). In mothers we stated significant positive correlation between IMT and TC concentration (Rs=0.831, p<0.01). In fathers there were positive correlations between IMT and Apo B (Rs=0.692, p<0.05), TG and sICAM-1 (Rs=0.912, p<0.01), TG and sE-selectin (Rs=0.678, p<0.05).

Lipid profile and concentration of apolipoproteins in newborns’ cord blood according to their birth weight is presented in Table 5. A significant correlation was found between body mass at birth and cord blood concentration of TG, VLDL and Apo B. Newborns with the lowest birth weight (2250-2500g), had the highest concentration of these parameters. In female newborns concentration of TC, LDL, HDL and Apo A1 was significantly higher than in male newborns (p<0.05). There were no correlation between sex and concentration of TG, VLDL and Apo B.

**Table 5:** Lipid and apolipoprotein concentrations (means ± SD) in newborns’ cord blood according to birth weight

Parameter	Birth weight (g)	Newborns (n)	Concentration	p
TG (mg/dl)	2250-2500	12	91.83±75.00	<0.05
	2501-3000	36	44.97±22.70	1:2
	3001-3500	44	62.39±94.38	1:3
	3501-4000	36	62.14±53.33	1:5
	4001-4500	9	38.44±6.48	4:5
TC (mg/dl)	2250-2500	12	70.67±3.44	ns
	2501-3000	36	63.36±27.27	
	3001-3500	44	66.29±18.94	
	3501-4000	36	63.14±18.98	
	4001-4500	9	65.89±20.73	
LDL (mg/dl)	2250-2500	12	34.41±10.30	ns
	2501-3000	36	33.45±17.94	
	3001-3500	44	35.84±13.19	
	3501-4000	36	32.39±11.61	
	4001-4500	9	34.96±12.19	
VLDL (mg/dl)	2250-2500	12	18.37±15.00	p<0.05
	2501-3000	36	9.00±4.58	1:5
	3001-3500	44	11.30±10.57	3:5
	3501-4000	36	12.75±10.58	4:5
	4001-4500	9	7.69±1.30	
HDL (mg/dl)	2250-2500	12	17.89±8.90	ns
	2501-3000	36	21.16±7.96	
	3001-3500	44	19.44±7.19	
	3501-4000	36	17.99±9.05	
	4001-4500	9	23.24±9.42	
Apo A1 (mg/dl)	2250-2500	12	89.17±10.02	ns
	2501-3000	36	90.14±20.41	
	3001-3500	44	90.25±18.80	
	3501-4000	36	89.11±14.35	
	4001-4500	9	85.89±29.25	
Apo B (mg/dl)	2250-2500	12	47.25±18.01	<0.05
	2501-3000	36	33.97±14.88	1:2
	3001-3500	44	35.48±12.82	
	3501-4000	36	41.47±22.27	
	4001-4500	9	39.33±20.24	

ns – non-significant

The mean maternal age was 26±3 years. In the study group, 64 (46.7%) women were in first pregnancy, 31 (22.6%) in their second pregnancy, 22 (16.1%) in third pregnancy and 20 (14.6%) in fourth or next pregnancy. There were also no significant differences between newborns lipids parameters and pregnancy order or gestational age. The body mass index (BMI) before pregnancy ranged between 20 and 24.9 kg/m^2^ in 62.8% of mothers. BMI lower than 19.9kg/m^2^ was reported by 22 women (16.1%), another 22 (16.1%) had BMI between 25 to 29.9 kg/m^2^ and 7 (5.1%) women were obese (BMI over 30 kg/m^2^). Table 6 presents newborns’ cord blood lipids parameters according to mothers BMI before pregnancy.

**Table 6:** Lipid and apolipoprotein concentrations (means ± SD) in newborns’ cord blood according to maternal pre-pregnancy BMI

Parameter	BMI (kg/m^2^)	Newborns (n)	M ±SD	p
TG (mg/dl)	<19.9	22	43.19±20.73	ns
	20-24.9	86	57.36±49.61	
	25-29.9	22	82.73±128.70	
	>30	7	47.14±13,80	
TC (mg/dl)	<19.9	22	59.00±17.37	ns
	20-24.9	86	65.57.22.27	
	25-29.9	22	71.09±21.53	
	>30	7	58.71±11.03	
LDL (mg/dl)	<19.9	22	31.78±13.91	ns
	20-24.9	86	34.32±14.11	
	25-29.9	22	37.16±14.62	
	>30	7	29.53±7.49	
VLDL (mg/dl)	<19.9	22	8.89±4.20	<0.05
	20-24.9	86	11.80±9.97	1:2
	25-29.9	22	13.32±13.73	
	>30	7	9.43±2.76	
HDL (mg/dl)	<19.9	22	18.90±5.18	ns
	20-24.9	86	19.55±9.46	
	25-29.9	22	20.61±7.16	
	>30	7	19.76±4.55	
Apo AI (mg/dl)	<19.9	22	87.05±10.80	<0.05
	20-24.9	86	88.30±20.08	1:3
	25-29.9	22	97.77±18.69	2:3
	>30	7	86.71±6.56	3:4
Apo B (mg/dl)	<19.9	22	31.55±6.73	<0.05
	20-24.9	86	39.64±20.51	1:2
	25-29.9	22	38.95±13.68	1:3
	>30	7	34.00±7.63	

ns – non-significant

## Discussion

The most current American Academy of Pediatrics (AAP) guidelines for cardiovascular health recommend to fasting lipid profile in children at increased risk for CVD for the first time between 2 and 10 years of age. In risk groups are children with: 1) a positive family history of dyslipidemia or premature (≤55 years of age for men and ≤65 years of age for women) CVD or dyslipidemia, 2) unknown family history, 3) other CVD risk factors, such as overweight (BMI ≥ 85th percentile, <95th percentile), obesity (BMI ≥95th percentile), hypertension (blood pressure ≥ 95th percentile), cigarette smoking, or diabetes mellitus [18].

The National Cholesterol Education Program (NCEP) Expert Panel on Blood Cholesterol Levels in Children and Adolescents specify positive family history in biological parents and grandparents for CVD as myocardial infarction, angina pectoris, peripheral or cerebral vascular disease, sudden death, coronary artery bypass-surgery or balloon angioplasty before age 55 [19]. It should be noted that in children CVD risk we evaluate medical history of first and second-degree relatives.

The most remarkable result of our study is significant increase of TG and VLDL serum concentration in children aged 1.5-2.5 years with a family history of premature CAD compared to controls. However we did not find any differences in lipid profile in newborns with a positive family history of premature CAD and controls.

Kelishadi et al. reported a significantly higher levels of TG, TC and LDL, and lower levels of HDL in children of parents with premature CAD [20]. Romaldini et al. showed hypercholesterolemia in 27.5% and hypertriglyceridemia in 12.8% of children of high-risk families [21]. In young adults aged 19 -30 years with parental premature CAD only TC concentration was significantly higher than in controls [22]. Marcovecchio et al. reported significant differences regarding TG and HDL between adolescents with a history of parental dyslipidemia and non-risk controls [23]. Mendes et al. revealed higher values of TC and lower of HDL in offspring of young adults with CAD than in controls [24]. It should be noted that our study was conducted only in children aged 1.5-2.5 years and most of mentioned studies were performed among older children and adolescents. Lipids and lipoproteins concentrations are the lowest during intrauterine life and at birth, and then increase until 2 years of age. Human lipid levels became quite constant up to adolescence [25]. Moreover, to understand some of these differences the ethnic and racial disparities should be also taken into consideration.

Elevated Lp(a) levels have been identified as independent risk factor for CAD that promotes atherogenesis and thrombogenesis [10, 26]. Several studies in children have found a positive association between Lp(a) level and positive family history of CVD [27–29]. This concept has been challenged by Tonstad et al. demonstrating that Lp(a) was marginally lower in the children with a history of premature cardiovascular death in male relatives (*p* = 0.033) [30]. In our study children, but not newborns, from kindreds with a history of premature CAD were also distinguished from controls by significantly higher Lp(a) levels. Since Lp(a) level is fully expressed in the first year of life, has a genetic adjustment, does not vary with age and, is almost unaltered by environmental factors, it should be widely applied to identification children at increased risk of CVD [28].

IMT is a well-established non-invasive marker of cardiovascular risk in adults. It has been showed that IMT >0.9 mm is related to significant atherosclerotic risk in adults [31]. In our study, the mean values of IMT in mothers and fathers did not exceed that cut-off level. Litwin et al. summarize growing body of evidence for increased IMT in children and adolescents with cardiovascular risk factors like obesity, hypertension or hyperlipidemia. It has been also reported that children with parental history of premature myocardial infarction have increased IMT independently of traditional atherosclerotic risk factors [32, 33]. However, it should be highlighted that age is the most important determinant of IMT, and increased IMT mostly was not seen in children under 10 years of age [31]. That fact may explain the lack of differences in IMT between children with a family history of premature CAD and controls in our study. Thus, efforts should be focused on enhance IMT measurements.

Several limitations to this study need to be acknowledged. The final sample size is quite small and there is no control group composed of adults. Moreover, no correction for multiple testing is a limitation. In the second stage of the study we do not evaluate impact of environmental cardiovascular risk factors like diet or physical activity on lipids parameters and IMT. It is suggested that the association of these factors is investigated in future studies. Further investigation, among the same subjects, to determine current cardiovascular risk is strongly recommended.

## Conclusions

The present study make several noteworthy contributions to atherosclerosis research. Measurement of serum Lp(a) level may be apply to identification cardiovascular risk in children and adolescents. IMT is not a reliable measure of atherosclerosis in the youngest children. Early detection of cardiovascular risk in children with a family history of premature cardiovascular diseases should result in prevention intervention in the entire family.

## Abbreviations

AAP: American Academy of Pediatrics
Apo A1: apolipoprotein A1
Apo B: apolipoprotein B
BMI: body mass index
CAD: coronary artery disease
CVD: cardiovascular disease
Hcy: homocysteine (Hcy)
HDL: high density lipoprotein
hs-CRP: high-sensitivity C-reactive protein
IMT: intima-media thickness
LDL: low density lipoprotein
Lp(a): lipoprotein a
NCEP: National Cholesterol Education Program
NHLBI: National Heart, Lung, and Blood Institute
oxy-LDL: oxidized low density lipoprotein
sICAM-1: soluble intercellular adhesion molecule-1
sVCAM-1: soluble vascular cell adhesion molecule-1
TC: total cholesterol
TG: triglycerides
VLDL: very low density lipoprotein
